# Effects of a diet supplemented with polysaccharides from *Pogostemon cablin* on growth performance, meat quality, and antioxidant capacity in Chongren Partridge chickens

**DOI:** 10.3389/fvets.2024.1381188

**Published:** 2024-05-27

**Authors:** Yantian Tang, Si Chen, Lingli Chen, Kehui Ouyang, Hui Chen, Wenjun Wang

**Affiliations:** ^1^College of Animal Science and Technology, Jiangxi Agricultural University, Nanchang, China; ^2^School of Life Science and Pharmacy, Jiujiang University, Jiujiang, China; ^3^Jiangxi Key Laboratory of Natural Products and Functional Food, College of Food Science and Engineering, Jiangxi Agricultural University, Nanchang, China

**Keywords:** *Pogostemon cablin*, polysaccharide, growth performance, meat quality, Chongren Partridge chicken

## Abstract

In this study, the *Pogostemon cablin* polysaccharides (PCPs) were heteropolysaccharides with molecular weights of 63.17 kDa and 8.99 kDa, and their total carbohydrate content was 76.17 ± 0.23%, uronic acid content was 19.92 ± 0.42%, and protein content was 1.24 ± 0.07%. PCP is composed of arabinose, galactose, glucose, and glucuronic acid, with a molar ratio of 0.196:0.249:0.451:0.104. In addition, we further investigated the effects of the diet supplemented with different doses of PCP on growth performance, meat quality, and anti-oxidant capacity in Chongren Partridge chickens. A total of 200 chickens were randomly allocated into 4 treatments, and fed with a basal diet of 0 (CON), 200 (LPCP), 400 (MPCP), and 800 (HPCP) mg/kg PCP for a 14-day prefeeding period and a formal experimental period of 56 days. Results showed that dietary PCP significantly increased final body weight (BW), average daily gain (ADG), and decreased feed-to-gain ratio (F/G) from days 1 to 56. Meanwhile, dietary PCP reduced yellowness (b^∗^) values and increased redness (a^∗^) values at 24 h in breast muscles (*p* < 0.05). Furthermore, LPCP and MPCP significantly increased the level of guanylic acid (GMP) (*p* < 0.05). MPCP increased the content of free amino acids (isoleucine, leucine, lysine, methionine, threonine, valine, alanine, glutamic acid, serine, cysteine), total essential amino acid (EAA), total flavor amino acid (FAA), total AA, the content of fatty acids (c14:1, c16:1, and c22:2), and monounsaturated fatty acids (MUFAs) in the breast muscle when compared to CON (*p* < 0.05). In addition, MPCP significantly reduced the content of malondialdehyde (MDA) and increased the transcript abundances of fatty acid desaturase 2 (*FADS2*), fatty acid synthase (*FAS*), lipoprotein lipase (*LPL*), and sterol regulatory element binding protein-1 (*SREBP-1*) in the breast muscles of the chickens (*p* < 0.05). In light of the aforementioned results, PCP at 400 mg/kg could be used as an effective additive because it not only promotes the growth performance of Chongren Partridge chickens but also shows a conducive role in meat quality, especially in meat flavor.

## 1 Introduction

In recent years, as consumer health awareness has increased, more attention has been given to producing safe, healthy, high-quality, nutritional meat. With the withdrawal of antibiotics from the breeding industry, pollution-free and non-residual polysaccharides were used as feed additives instead of antibiotics to produce efficient and green livestock products, which have attracted a great deal of interest in animal breeding (1–3). Polysaccharides, a kind of biological macromolecule with complex structures and a wide range of biological activities, exist widely in the cell membrane of plants and animals, as well as the cell wall of microorganisms (bacteria and fungi) (4). Polysaccharides possess a variety of beneficial properties, such as antioxidant, hypolipidemic, hypoglycemic, and anti-tumor (5–7). Meanwhile, polysaccharides can regulate intestinal microecology and improve the immunity of animal (8).

*Pogostemon cablin* (Blanco) Benth is a traditional Chinese medicine and food homologous plant. The stems and leaves of *Pogostemon cablin* are rich in essential oils and aromatic compounds and have anti-inflammatory and antibacterial activities. It has been shown that pogostone has a protective effect against LPS-induced acute lung injury in mice by regulating the Keap1-Nrf2/NF-κB signaling pathway (9). Patchoulene epoxide isolated from patchouli oil could inhibit acute inflammation by blocking the NF-κB signaling pathway and inhibiting the activation of iNOS and COX-2 (10). The water extracts of *Pogostemon cablin* inhibit the activity of xanthine oxidase (11). In addition, the study has shown that *Pogostemon cablin* polysaccharides (PCPs) have antiviral activities against the porcine epidemic diarrhea virus (12).

However, the effects of PCPs on meat quality in animals have not been reported. Therefore, the purpose of this study was to study the effects of diets supplemented with PCP on the growth performance, antioxidant status, and meat quality of Chongren Partridge chickens, thereby providing the theoritical basis for its application in broiler production.

## 2 Materials and methods

### 2.1 Preparation of PCP

*Pogostemon cablin* was added to 95% ethanol and allowed to stand at room temperature for 12 h. It was rinsed with water, dried at 60°C and weighed. The sample was added to 20 times the volume of distilled water and extracted twice at 90°C for 2 h. The extract was mixed with 95% ethanol to concentrate and precipitate. Proteins were removed by Sevage method, dialyzed, and freeze-dried to obtain PCP.

### 2.2 Characterization of PCP

#### 2.2.1 Determination of molecular weight

The molecular weight was determined using high-performance gel permeation chromatography. The chromatographic column was a BRT105-104-102 tandem gel column (8 mm × 300 mm) with an RI-10A differential detector. The mobile phase was 0.05 M NaCl solution, the flow rate was 0.6 mL/min, the column temperature was 40°C, and the injection volume was 20 μL.

#### 2.2.2 Chemical composition

Total carbohydrate content was determined by the phenol-sulfuric acid method, protein content was determined by the Thomas Brilliant Blue method, and glucuronic acid content was determined by the m-hydroxybiphenyl method (13).

#### 2.2.3 Analysis of monosaccharides composition

GC–MS (QP2010, Shimadzu, Japan) was used to analyze the monosaccharide composition of PCP with appropriate modifications. Briefly, the monosaccharide composition of PCP was identified and quantified by GC–MS on a Dionex Carbopac™pa20 column (3 μm × 150 mm). The carrier gas was helium, the flow rate was 1 mL/min, the temperature of the injector was 250°C, and the column temperature was increased from 120°C to 250°C at a rate of 3/min and kept for 5 min.

### 2.3 Animals and diets

The experimental design was accepted by the Ethics Committee of the Local Experimental Animals Care Committee and performed under the guidelines of the Animal Care and Welfare Committee of Jiangxi Agricultural University (JXAUA1101). A total of 200 one-day-old female Chongren Partridge chickens with similar initial body weights (BWs) were randomly assigned to 4 groups with 5 replicates containing 10 chickens per replicate for a 14-day prefeeding period and a formal experimental period of 56 days. The basal diet was formulated to meet the nutrient requirements of Chongren Partridge chickens according to the broiler nutrient requirements (Table 1). Dietary treatments consisted of the basal diet supplemented with 0 (CON), 200 (LPCP), 400 (MPCP), and 800 (HPCP) mg/kg polysaccharides from *Pogostemon cablin*. Chickens had free access to feed and water throughout the 56-day experimental period; daylight was eliminated and replaced with 18-h lighting from incandescent bulbs and room temperature at 26°C.

**Table 1:** Composition and nutrient levels of diets (as-fed basis).

Items	1 to 28 d	29 to 56 d
Ingredients, %		
Corn	55.56	58.01
Soybean oil	4.48	5.81
Soybean meal	31	28.58
Fish meal	4.45	3.62
Limestone	1.45	1.10
CaHPO4	1.12	1.24
Methionine	0.21	0.11
Lysine	0.14	0.10
choline chloride	0.29	0.13
NaCl	0.3	0.3
Premix^1^	1	1
Nutrient Levels		
ME, MJ/kg	12.67	12.82
Crude Protein, %	21.24	20.01
Crude Fiber, %	3.12	3.02
Crude Fat, %	7.06	8.42
Calcium, %	0.98	0.82
Available phosphorus, %	0.40	0.41
Lysine, %	1.28	1.18
Methionine + Cysteine, %	0.81	0.72

^1^The premix provided following per kilogram of basal diet: vitamin A 10000 IU; vitamin D3 1,000 IU; vitamin E 50 IU; vitamin K 1.1 mg; vitamin B12 20 mg; pantothenic acid 50 mg; nicotinic acid 200 mg; folic acid 20 mg; biotin 0.5 mg; Zn 70 mg; Fe 100 mg; Mn 55 mg; Cu 9 mg; I 0.3 mg; Se 0.15 mg.

### 2.4 Growth performance

Chickens were weighted at 1 and 56 days of the experiment, and daily gain, feed intake was recorded daily per pen. The final BW, average daily gain (ADG), average daily feed intake (ADFI), and feed-to-gain ratio (F/G) were calculated at the end of the experiment.

### 2.5 Carcass traits

On day 56 of the experiment, 2 birds with the average BW from each replicate were randomly selected and slaughtered. The liver, spleen, thymus, and bursa of Fabricius were dissected and weighed. Relative weight of immune organs = the immune organ weight/live BW × 100%.

### 2.6 Meat quality

On day 56 of the experiment, 2 birds with the average BW from each replicate were randomly selected and slaughtered. At 45 min and 24 h post-mortem, the pH and meat color (L^*^, a^*^, and b^*^) were measured using a portable pH meter (Testo-205, Lenzkirch, Germany) and a color meter (Chroma Meter CR-410, Chiyoda, Japan), respectively. Shear force was assessed with the digital muscle tenderness tester (C-LM3B, TENOVO, China). The samples of breast muscles were cooked in 80°C water to an internal temperature of 70°C and then cooled to room temperature. Cooked samples were sheared perpendicular to the fiber orientations, and the average peak shear force was measured.

### 2.7 Meat composition

Crude protein was measured using an automatic nitrogen analyzer (FOSS 8400, Hillerod, Denmark). Intramuscular fat (IMF) was determined using a fat analyzer (FOSS 2055, Hillerod, Denmark). Inosine monophosphate (IMP) and guanosine monophosphate (GMP) were entrusted to Nanchang Customs District, P. R. China for measurement using high-performance liquid chromatography (HPLC).

### 2.8 Free amino acids

Free amino acids (FAAs) of deproteinized muscles were measured by an automatic amino acid analyzer (Hitachi L-8900, Tokyo, Japan). Approximately 1 g meat sample was homogenized and deproteinized using 20% sulfosalicylic acid (3 mL), then centrifuged for 15 min at 12,000 × g at 4°C, and the supernatant was filtered by 0.22 μm filter for FAA determination.

### 2.9 Fatty acid composition

Fatty acids were calculated according to the determination of fatty acids in food (GB5009. 168–2016, China). Muscle samples (10 g) were mixed with 2 mL of undecanoic acid, 1,1′,1″-(1,2,3-propanetriyl) ester solution, and 10 mL of hydrochloric acid solution, and hydrolyzed for 40 min at 70°C to 80°C water bath. After cooling, the hydrolysate obtained by 10 mL of 95% ethyl alcohol was added and extracted three times with ether–petroleum ether mixture. The crude fat extract was obtained using a rotary evaporator. Then, saponification and methylation were performed on fatty acid methyl esters in alkaline conditions. The gas chromatography conditions were set as follows: the injector and detector temperature were held at 270°C and 280°C; the initial column temperature was kept at 100°C for 13 min, increased to 180°C at 10°C/min, and kept at 180°C for 6 min; the temperature was increased to 200°C at 1°C/min and maintained at 200°C for 20 min; and then the temperature was increased to 230°C and held at 230°C for 10.5 min. Finally, fatty acids were identified using a hydrogen flame ion detector.

### 2.10 Antioxidant capability

The breast muscle samples were homogenized in 0.9% NaCl at 1:9 (m:v) and centrifuged for 15 min at 3500 × g at 4°C. The supernatant was then collected. Total antioxidant capacity (T-AOC), glutathione peroxidase (GSH-Px), total superoxide dismutase (T-SOD) activity, and malondialdehyde (MDA) contents were measured using the appropriate kits according to the manufacturer’s protocols (Nanjing Jiancheng Bioengineering Institute, Nanjing, China).

### 2.11 RNA extraction and quantitative RT-PCR

Total RNA was extracted from the breast muscle samples using TRIzol reagent (TaKaRa, Dalian, China) and then reverse-transcribed into cDNA according to the manufacturer’s instructions (TaKaRa). Real-time quantitative PCR was performed using the primers listed in Table 2. The relative mRNA level was calculated by the 2^−ΔΔCt^ method with *β-actin* mRNA as an internal control.

**Table 2:** Primers used in the real-time quantitative PCR.

Gene name	Primer sequences (5′-3′)	Accession ID
*β-actin*	Forward: CGGACTGTTACCAACACCCA Reverse: ATCCTGAGTCAAGCGCCAAA	NM_205518.2
*FADS1*	Forward: CCTCTTCTCTGCGTTGCTTC Reverse: ATTTGTGCACCCAGTGGTTC	HQ667600.1
*FADS2*	Forward: CATCTTCCCACCTCTGCTCA Reverse: GCGCATGTAGTAGCTGATGG	NM_001160428.3
*ELOVL2*	Forward: CCATGTGGGTTTCCCTTTGG Reverse: TGCAGCTGTTCTTGAAGGTG	NM_001197308.2
*ELOVL5*	Forward: AGCTACCTGGATGTTTGGCT Reverse: AAGCAGTGTGAGTCCAAGGT	NM_001199197.2
*FAS*	Forward: AGAAGCTGCTGAAGCCCTTA Reverse: CTGTCCGTGACGAATTGCTT	NM_205155.4
*LPL*	Forward: GGGTCCCAAAGCTAGTGGAT Reverse: GTGTAAGCAGCAGACACTGG	EU477529.1
*HMGCR*	Forward: GTCTTGTGATTGGCGTTGGT Reverse: ACGTCCTTCACGACTCTCTC	NM_204485.3
*CPT1α*	Forward: TAGGACCAAGGCTTCAGTGG Reverse: AGCTCTAGCTGCCTGTATGG	NM_001012898.1
*SREBP1*	Forward: TACCGCTCATCCATCAACGA Reverse: TTCAGGCTGAGGTTCTCCTG	NM_204126.3

*FADS1*, Fatty acid desaturase 1; *FADS2*, fatty acid desaturase 2; *ELOVL2*, fatty acid elongase 2; *ELOVL5*, fatty acid elongase 5; *FAS*, fatty acid synthase; *LPL*, lipoprotein lipase; *HMGCR*, 3-hydroxy-3-methylglutaryl-coenzyme A reductase; *CPT1α*, carnitine palmitoyltransferase-1α; *SREBP-1*, sterol regulatory element binding protein-1.

### 2.12 Statistical analysis

All the data were analyzed by one-way analysis of variance (ANOVA) using Duncan’s multiple range tests using SPSS 20.0 for Windows. Results were expressed as mean and SEM, and statistical significance was set at a *p*-value of ≤0.05.

## 3 Results

### 3.1 Physicochemical properties and structure of PCP

**Figure 1:**
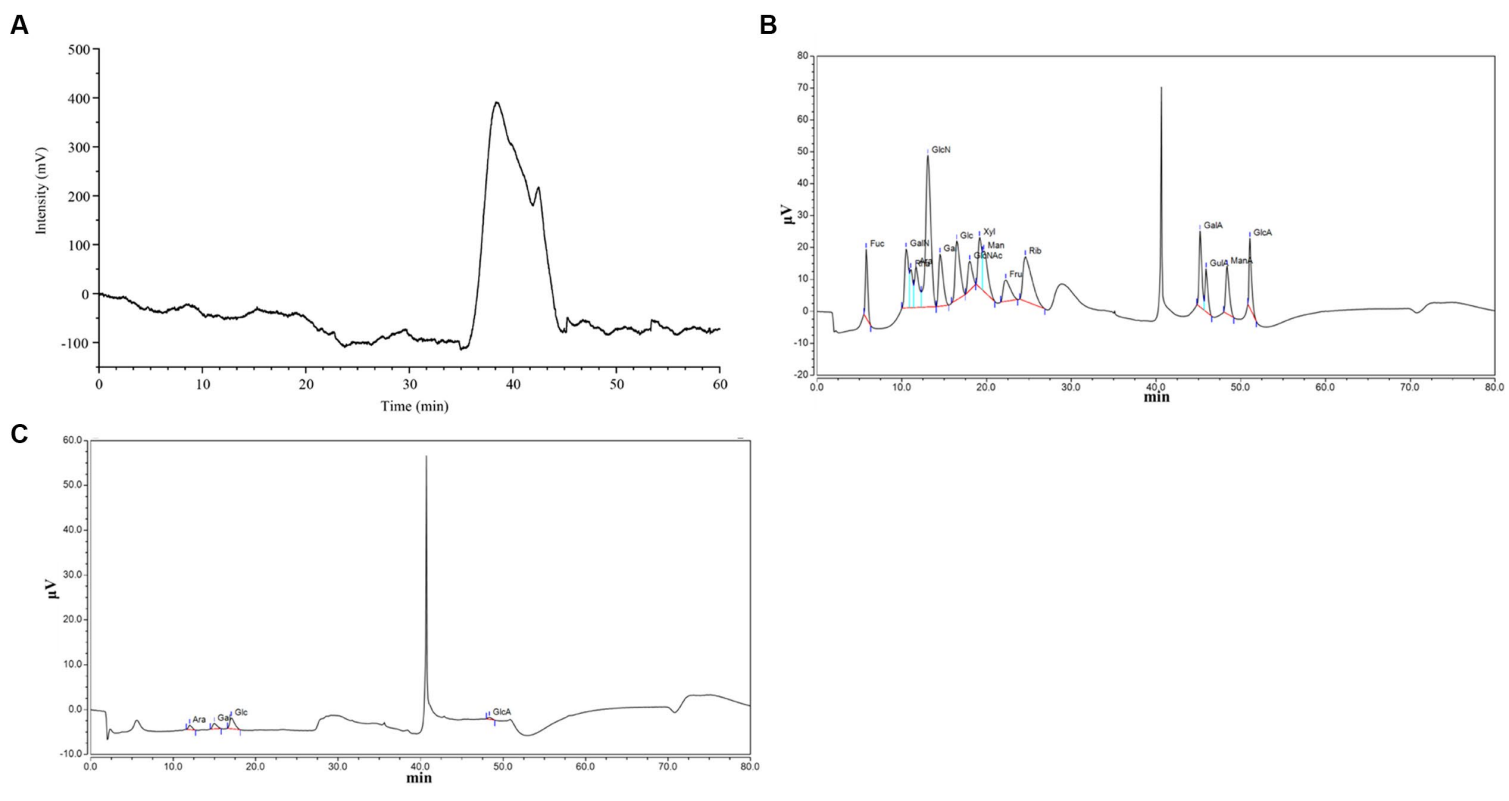
Structure of CPP20. **(A)** Molecular weight; **(B,C)** monosaccharide composition.

The physicochemical properties and structures of PCP are shown in Table 3 and Figure 1. PCP was a heteropolysaccharide with a molecular weight of 63.17 kDa and 8.99 kDa, and its total carbohydrate content was 76.17 ± 0.23%, uronic acid content was 19.92 ± 0.42%, and protein content was 1.24 ± 0.07%. PCP is composed of arabinose, galactose, glucose, and glucuronic acid, with a molar ratio of 0.196:0.249:0.451:0.104.

**Table 3:** Chemical composition of PCP.

Sample	Total carbohydrate (%)	Uronic acid (%)	Protein (%)
PCP	76.17 ± 0.23%	19.92 ± 0.42%	1.24 ± 0.07%

### 3.2 Growth performance

The growth performance of chickens is presented in Table 4. Diets supplemented with PCP significantly improved FBW and ADG and decreased F/G from days 1 to 56 compared to the control diet (*p* < 0.05). However, IBW and ADFI from days 1 to 56 were not significantly different among LPCP, MPCP, HPCP, and CON diets (*p* > 0.05).

**Table 4:** Effects of dietary PCP on the growth performance of Chongren Partridge chickens.

Items	CON	LPCP	MPCP	HPCP	*p*-value
IBW, g	105.02 ± 0.26	105.22 ± 0.12	105.28 ± 0.14	105.06 ± 0.28	0.791
FBW, g	1284.14 ± 13.05^b^	1347.22 ± 10.08^a^	1394.68 ± 11.92^a^	1375.68 ± 33.59^a^	0.006
ADG, g	21.06 ± 0.24^b^	22.18 ± 0.18^a^	23.03 ± 0.21^a^	22.69 ± 0.60^a^	0.006
ADFI, g	65.98 ± 0.48	66.18 ± 0.42	66.89 ± 0.48	66.95 ± 1.35	0.755
F/G	3.13 ± 0.03^a^	2.98 ± 0.02^b^	2.91 ± 0.03^b^	2.95 ± 0.03^b^	<0.001

Values are expressed as the mean ± SEM (*n* = 10). ^a,b^Means in the same row with different superscripts differ significantly (*p* ≤ 0.05). IBW, Initial body weight; FBW, final body weight; ADG, average daily gain; ADFI, average daily feed intake; F/G, feed/gain ratio; CON, control; LPCPs, low-dose polysaccharides extracted from *Pogostemon cablin*; MPCPs, medium-dose polysaccharides extracted from *Pogostemon cablin*; HPCPs, high-dose polysaccharides extracted from *Pogostemon cablin*.

### 3.3 Relative weight of immune organs

Immune organs’ relative weight of chickens is shown in Table 5. Compared with the control group, dietary PCP did not affect the relative weight of organs such as liver, spleen, thymus, and bursa (*p* > 0.05).

**Table 5:** Effects of dietary PCP on immune organs relative weight of Chongren Partridge chickens at 56 d (g/kg of body weight).

Items	CON	LPCP	MPCP	HPCP	*p*-value
Liver	2.01 ± 0.043	1.92 ± 0.079	1.88 ± 0.069	1.98 ± 0.048	0.532
Spleen	0.14 ± 0.007	0.14 ± 0.004	0.13 ± 0.006	0.18 ± 0.013	0.084
Thymus	0.03 ± 0.002	0.03 ± 0.002	0.04 ± 0.004	0.03 ± 0.004	0.637
Bursa	0.21 ± 0.007	0.23 ± 0.013	0.17 ± 0.018	0.22 ± 0.029	0.123

Values are expressed as the mean ± SEM (*n* = 10). ^a,b^Means in the same row with different superscripts differ significantly (*p* ≤ 0.05). CON, Control; LPCPs, low-dose polysaccharides extracted from *Pogostemon cablin*; MPCPs, medium-dose polysaccharides extracted from *Pogostemon cablin*; HPCPs, high-dose polysaccharides extracted from *Pogostemon cablin*.

### 3.4 Meat quality

The meat quality of breast muscle samples is illustrated in Table 6. Dietary PCP significantly decreased the b^*^ value at 45 min and 24 h and increased the a^*^ value at 24 h compared with the control (*p* < 0.05). HPCP decreased the L^*^ value compared to CON, while HPCP had a greater a^*^ value than LPCP at 45 min (*p* < 0.05).

**Table 6:** Effects of dietary PCP on meat quality of Chongren Partridge chickens at 56 days.

Items	CON	LPCP	MPCP	HPCP	*p*-value
L*45 min	55.38 ± 0.43^a^	53.49 ± 0.65^ab^	53.23 ± 0.80^ab^	51.92 ± 0.96^b^	0.001
a*45 min	3.02 ± 0.16^ab^	1.91 ± 0.44^b^	2.74 ± 0.36^ab^	3.73 ± 0.41^a^	0.004
b*45 min	16.16 ± 0.36^a^	12.97 ± 0.29^c^	14.27 ± 0.46^bc^	14.56 ± 0.56^b^	0.001
L*24 h	51.04 ± 0.35^a^	49.46 ± 0.26^b^	49.28 ± 0.39^b^	50.54 ± 0.41^ab^	0.005
a*24 h	2.38 ± 0.08^b^	3.12 ± 0.12^a^	2.97 ± 0.08^a^	3.17 ± 0.10^a^	0.001
b*24 h	11.63 ± 0.18^a^	10.35 ± 0.19^bc^	9.71 ± 0.14^c^	10.52 ± 0.19^b^	0.001
pH45 min	5.88 ± 0.07	5.91 ± 0.05	5.82 ± 0.04	5.88 ± 0.04	0.710
pH24 h	6.12 ± 0.13	5.91 ± 0.09	5.93 ± 0.15	6.06 ± 0.33	0.829
Shear force, *N*	25.15 ± 0.41	23.36 ± 0.63	23.52 ± 0.84	24.66 ± 0.96	0.288

Values are expressed as the means ± SEM (*n* = 10). ^a,b^Means in the same row with different superscripts differ significantly (*p* ≤ 0.05). CON, Control; LPCPs, low-dose polysaccharides extracted from *Pogostemon cablin*; MPCPs, medium-dose polysaccharides extracted from *Pogostemon cablin*; HPCPs, high-dose polysaccharides extracted from *Pogostemon cablin*; L^*^, lightness; a^*^ = redness; b^*^ = yellowness.

### 3.5 Muscle chemical composition

**Figure 2:**
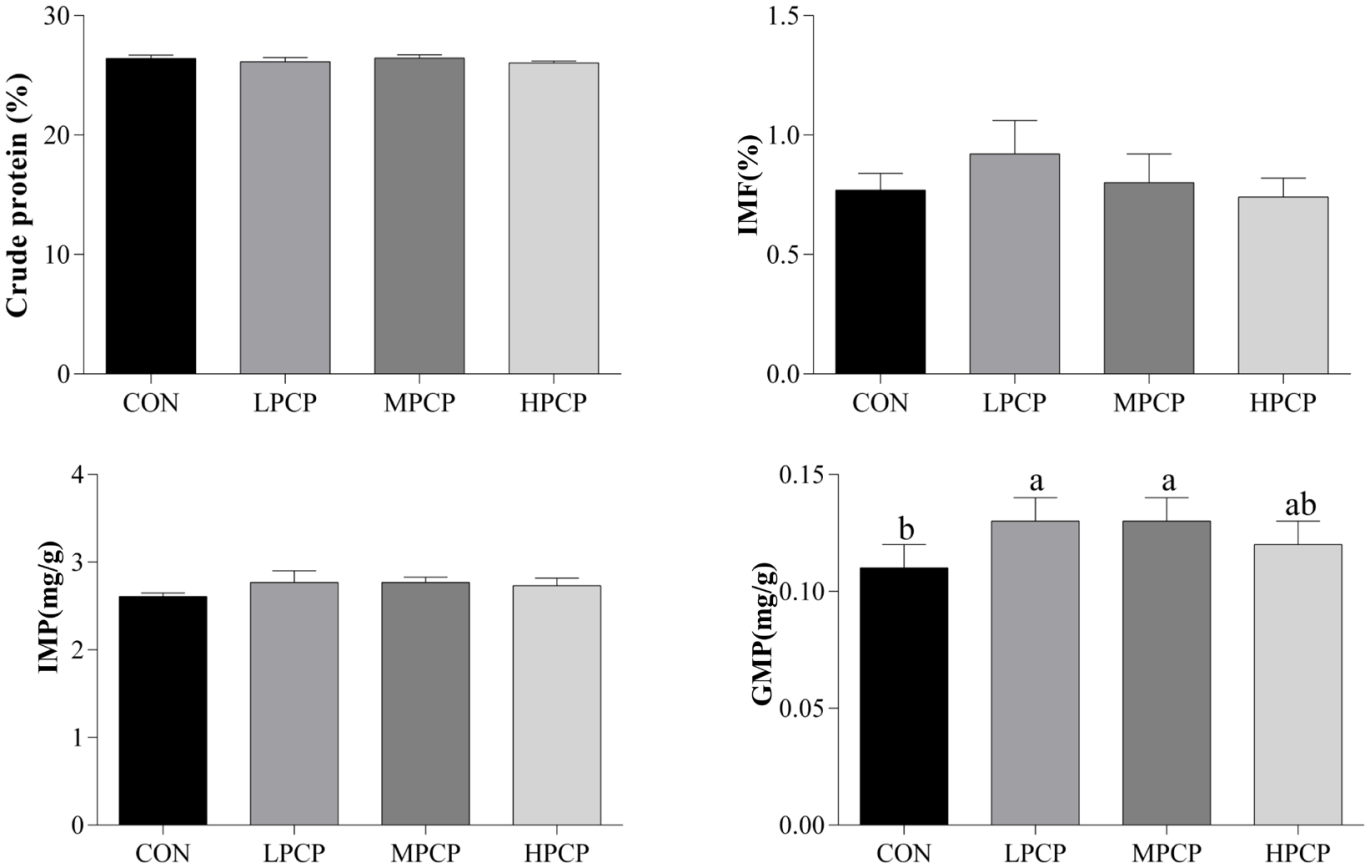
Effects of dietary PCP supplementation on muscle chemical composition of Chongren Partridge chickens at 56 days. Values are expressed as the means ± SEM (*n* = 10). ^a,b^ Means in the same row with different superscripts differ significantly (*p* ≤ 0.05). CON, Control; LPCPs, low-dose polysaccharides extracted from *Pogostemon cablin*; MPCPs, medium-dose polysaccharides extracted from *Pogostemon cablin*; HPCPs, high-dose polysaccharides extracted from *Pogostemon cablin*; IMF, intramuscular fat; IMP, inosinic acid; GMP, guanylic acid.

The muscle chemical composition of breast muscle samples is presented in Figure 2. LPCP and MPCP significantly increased the content of GMP compared to control (*p* < 0.05). There were no significant differences in crude protein, IMF, or IMP among the four diets (*p* > 0.05).

### 3.6 Amino acid profile

The amino acid profile of breast muscle samples is shown in Table 7. Compared with CON, LPCP decreased the content of free histidine but increased the content of free methionine and cysteine (*p* < 0.05). MPCP increased the content of free isoleucine, leucine, lysine, methionine, threonine, valine, alanine, glutamic acid, serine, cysteine, total EAA, total FAA, and total AA compared to CON (*p* < 0.05). HPCP increased free valine compared to CON (*p* < 0.05). In addition, the content of free histidine and valine in breast muscles was higher in MPCP and HPCP than in LPCP (*p* < 0.05). The content of free isoleucine, threonine, alanine, glutamic acid, total EAA, total FAA, and total AA was higher in MPCP than those in LPCP and HPCP (*p* < 0.05), but there were no significant differences between LPCP and HPCP (*p* < 0.05). In HPCP, the content of free lysine and cysteine was lower than those in MPCP (*p* < 0.05); meanwhile, the content of free methionine and cysteine in LPCP was higher than those in HPCP (*p* < 0.05).

**Table 7:** Effects of dietary PCP on the free amino acid profile of Chongren Partridge chickens at 56 days (mg/100 g meat).

Items	CON	LPCP	MPCP	HPCP	*p-*value
Arginine	7.84 ± 0.85	9.78 ± 0.82	10.77 ± 1.11	8.47 ± 0.65	0.207
Histidine	406.03 ± 2.99^a^	384.56 ± 5.06^b^	410.29 ± 5.31^a^	401.68 ± 5.54^a^	0.023
Isoleucine	6.98 ± 0.42b	6.84 ± 0.94b	11.16 ± 1.23a	7.85 ± 0.80b	0.004
Leucine	13.17 ± 0.70^b^	15.21 ± 2.12^ab^	18.85 ± 1.76^a^	16.19 ± 1.46^ab^	0.028
Lysine	13.53 ± 0.76^b^	18.05 ± 1.94^ab^	19.26 ± 2.49^a^	13.76 ± 1.09^b^	0.021
Methionine	6.71 ± 0.32^c^	8.47 ± 0.28^a^	8.05 ± 0.49^ab^	7.26 ± 0.56^bc^	0.02
Phenylalanine	7.02 ± 0.33	7.56 ± 0.27	7.88 ± 0.82	7.22 ± 0.12	0.108
Threonine	8.45 ± 0.51^b^	9.80 ± 0.57^b^	12.94 ± 0.87^a^	8.47 ± 0.85^b^	0.003
Valine	7.14 ± 0.61^b^	7.14 ± 0.74^b^	11.13 ± 0.45^a^	8.76 ± 1.01^a^	0.015
Alanine	16.84 ± 1.23^b^	19.65 ± 1.11^b^	36.80 ± 2.24^a^	20.33 ± 2.51^b^	0.000
Aspartic acid	8.58 ± 0.27	8.62 ± 0.68	8.87 ± 0.74	8.46 ± 0.49	0.952
Glutamic acid	31.58 ± 1.33^b^	30.03 ± 1.71^b^	47.38 ± 2.07^a^	34.12 ± 2.27^b^	<0.001
Glycine	10.57 ± 0.51	11.79 ± 0.99	12.75 ± 0.24	10.67 ± 0.56	0.158
Serine	26.49 ± 1.25^b^	27.90 ± 1.39^ab^	33.09 ± 1.11^a^	27.52 ± 1.25^ab^	0.065
Tyrosine	8.39 ± 0.86	8.16 ± 0.33	9.54 ± 0.87	8.01 ± 0.33	0.149
Cysteine	18.22 ± 1.12^b^	25.48 ± 0.39^a^	25.04 ± 0.07^a^	19.88 ± 1.79^b^	0.001
Total EAA	475.96 ± 4.61^b^	467.55 ± 9.24^b^	510.81 ± 10.02^a^	479.67 ± 10.43^b^	0.24
Total FAA	77.89 ± 2.37^b^	79.95 ± 2.87^b^	116.57 ± 3.15^a^	82.06 ± 5.17^b^	<0.001
Total AA	602.79 ± 10.75^b^	599.27 ± 12.44^b^	684.6 ± 11.89^a^	608.67 ± 17.04^b^	0.002

Values are expressed as the means ± SEM (*n* = 6). ^a,b^Means in the same row with different superscripts differ significantly (*p* ≤ 0.05). CON, Control; LPCPs, low-dose polysaccharides extracted from *Pogostemon cablin*; MPCPs, medium-dose polysaccharides extracted from *Pogostemon cablin*; HPCPs, high-dose polysaccharides extracted from *Pogostemon cablin*; EAA, essential amino acid; FAA, flavor amino acid; AA, amino acid.

### 3.7 Fatty acid composition

The fatty acid composition of breast muscle samples is presented in Table 8. The content of c14:1, c16:1, and total MUFA in MPCP was higher among the four diets (*p* < 0.05), and the content of c22:2 in MPCP was higher than that in CON (*p* < 0.05). Additionally, no significant effects were observed in the total content of SFA, PUFA, and UFA/SFA, as well as the content of other fatty acids except for c14:1, c16:1, and c22:2 among four diets (*p* > 0.05).

**Table 8:** Effects of dietary PCP supplementation on fatty acid composition of Chongren Partridge chickens at 56 days (% total fatty acids).

Items	CON	LPCP	MPCP	HPCP	*p*-value
c11:0	0.07 ± 0.02	0.07 ± 0.02	0.1 ± 0.02	0.05 ± 0.01	0.356
c12:0	0.23 ± 0.06	0.36 ± 0.08	0.46 ± 0.07	0.25 ± 0.06	0.236
c13:0	0.02 ± 0.01	0.04 ± 0.01	0.04 ± 0.01	0.02 ± 0.01	0.382
c14:0	1.07 ± 0.42	0.80 ± 0.25	1.36 ± 0.26	1.07 ± 0.38	0.668
c14:1	0.78 ± 0.42^b^	0.78 ± 0.11^b^	1.76 ± 0.16^a^	0.98 ± 0.13^b^	0.005
c15:0	0.17 ± 0.06	0.16 ± 0.04	0.24 ± 0.03	0.16 ± 0.05	0.288
c15:1	0.02 ± 0.01	0.03 ± 0.01	0.05 ± 0.01	0.02 ± 0.01	0.134
c16:0	28.27 ± 8.97	23.98 ± 8.68	32.79 ± 6.26	23.73 ± 6.66	0.777
c16:1	1.39 ± 0.07^b^	1.52 ± 0.17^b^	4.94 ± 0.73^a^	1.66 ± 0.61^b^	0.004
c17:0	0.17 ± 0.06	0.13 ± 0.03	0.20 ± 0.03	0.15 ± 0.03	0.532
c17:1	0.04 ± 0.01	0.07 ± 0.01	0.07 ± 0.01	0.04 ± 0.01	0.279
c18:0	7.88 ± 1.44	6.78 ± 0.79	10.90 ± 1.38	7.28 ± 1.30	0.233
c18:1	30.73 ± 11.56	21.59 ± 2.87	33.21 ± 3.48	30.63 ± 6.04	0.584
c18:2	24.3 ± 2.80	26.02 ± 4.09	31.96 ± 2.58	26.18 ± 3.58	0.365
c20:0	0.20 ± 0.05	0.21 ± 0.04	0.24 ± 0.02	0.23 ± 0.02	0.689
c20:1	0.53 ± 0.19	0.35 ± 0.12	0.62 ± 0.10	0.34 ± 0.08	0.305
c20:2	0.24 ± 0.08	0.18 ± 0.04	0.21 ± 0.03	0.15 ± 0.03	0.576
c20:3n6	6.90 ± 1.35	6.10 ± 1.00	6.58 ± 0.64	5.67 ± 0.82	0.826
c20:4	0.21 ± 0.04	0.20 ± 0.03	0.24 ± 0.04	0.15 ± 0.04	0.416
c21:0	0.30 ± 0.08	0.33 ± 0.11	0.39 ± 0.06	0.21 ± 0.03	0.351
c20:5	17.67 ± 3.79	15.98 ± 2.89	22.15 ± 2.57	13.71 ± 2.1	0.188
c22:0	1.34 ± 0.21	2.80 ± 1.30	2.19 ± 0.33	1.81 ± 0.44	0.623
c22:1	0.18 ± 0.01	0.17 ± 0.01	0.19 ± 0.02	0.17 ± 0.03	0.859
c22:2	0.05 ± 0.01^b^	0.11 ± 0.01^ab^	0.21 ± 0.02^a^	0.12 ± 0.02^ab^	0.016
c23:0	0.45 ± 0.08	0.36 ± 0.12	0.35 ± 0.03	0.29 ± 0.03	0.423
c22:6	1.11 ± 0.63	0.56 ± 0.13	0.87 ± 0.28	0.66 ± 0.10	0.793
SFA	46.34 ± 1.59	45.39 ± 1.35	45.98 ± 3.29	39.52 ± 1.61	0.616
MUFA	53.52 ± 3.44^b^	46.77 ± 3.37^b^	73.78 ± 1.44^a^	50.61 ± 1.79^b^	0.003
PUFA	35.42 ± 4.92	39.87 ± 4.32	53.75 ± 1.33	36.02 ± 1.81	0.061
MUFA/SFA	1.16 ± 0.07	1.10 ± 0.10	1.04 ± 0.12	0.96 ± 0.05	0.183

Values are expressed as the means ± SEM (*n* = 6). ^a,b^Means in the same row with different superscripts differ significantly (*p* ≤ 0.05). CON, Control; LPCPs, low-dose polysaccharides extracted from *Pogostemon cablin*; MPCPs, medium-dose polysaccharides extracted from *Pogostemon cablin*; HPCPs, high-dose polysaccharides extracted from *Pogostemon cablin*; SFAs, saturated fatty acids; MUFAs, monounsaturated fatty acids; PUFAs, polyunsaturated fatty acids.

### 3.8 Antioxidant capability of breast muscles

**Figure 3:**
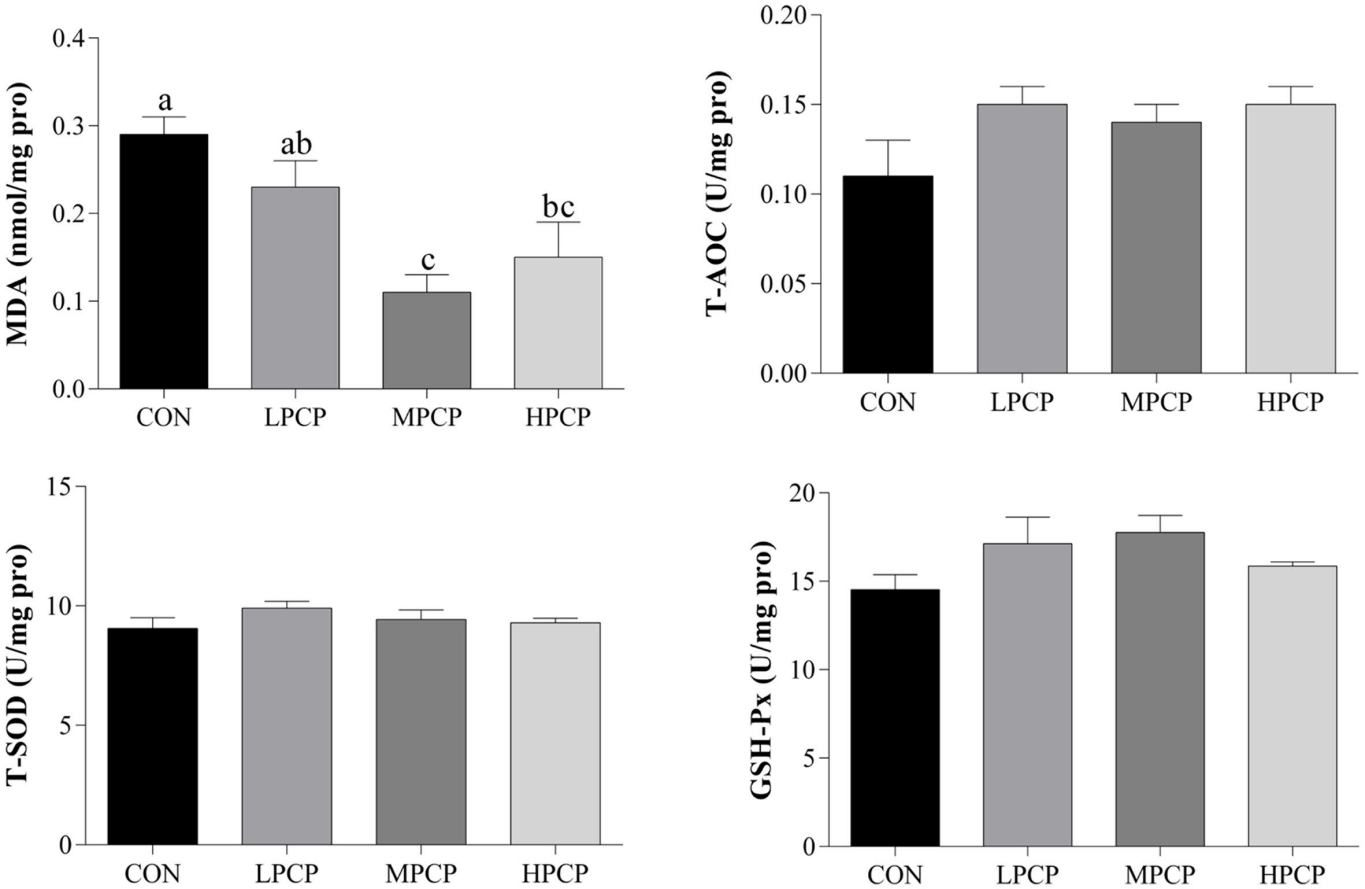
Effects of dietary PCP supplementation on the antioxidant capability of breast muscles in Chongren Partridge chickens at 56 days. Values are expressed as the means ± SEM (*n* = 6). ^a,b^ Means in the same row with different superscripts differ significantly (*p* ≤ 0.05). CON, Control; LPCPs, low-dose polysaccharides extracted from *Pogostemon cablin*; MPCPs, medium-dose polysaccharides extracted from *Pogostemon cablin*; HPCPs, high-dose polysaccharides extracted from *Pogostemon cablin*.

The antioxidant capability of breast muscle samples is shown in Figure 3. Compared with CON, MPCP and HPCP decreased the MDA content (*p* < 0.05). However, there were no significant differences in T-AOC, T-SOD, or GSH-Px among the four diets (*p* > 0.05).

### 3.9 Transcript expression of genes related to lipid metabolism

**Figure 4:**
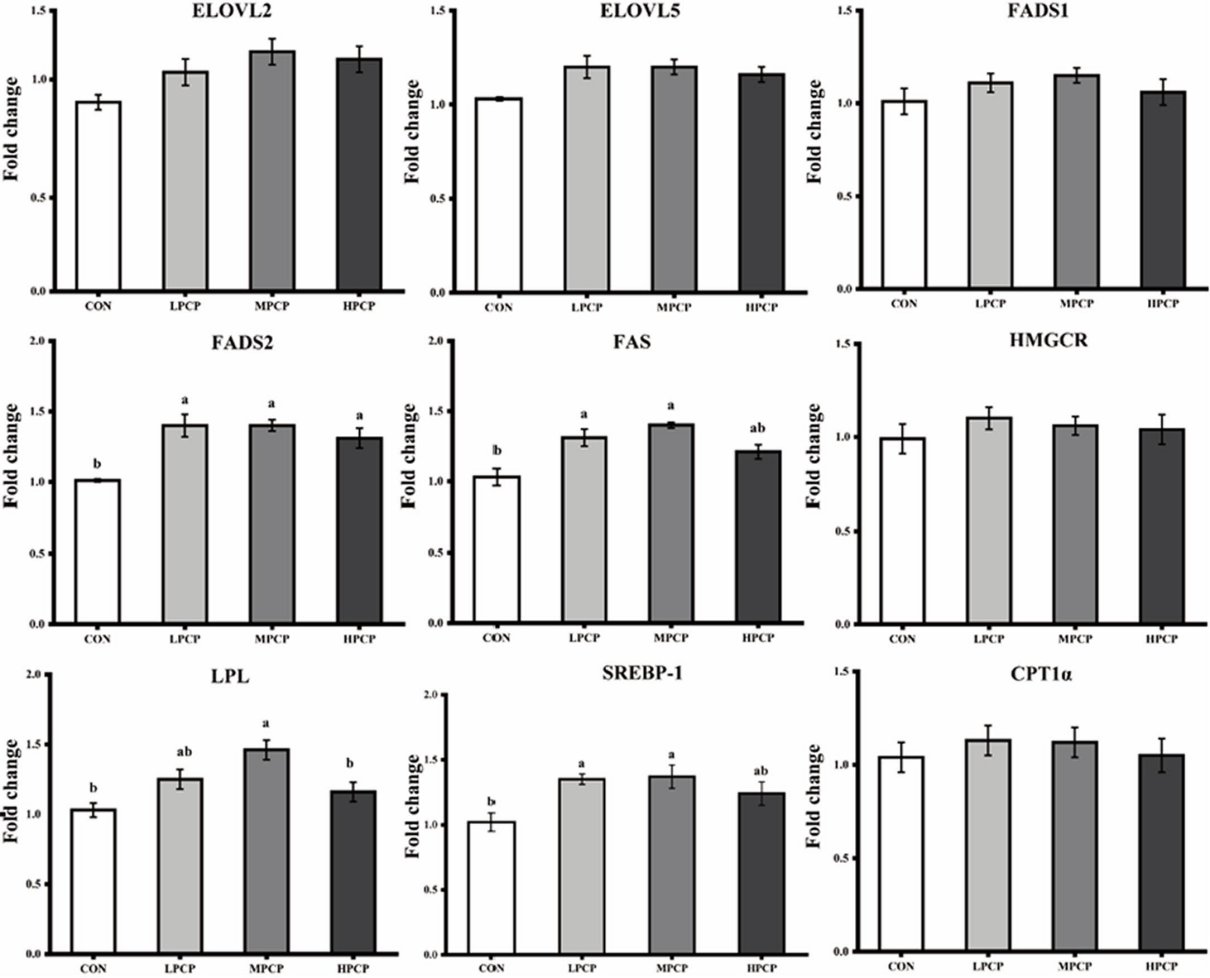
Effects of dietary PCP on the relative mRNA expression of genes related to lipid metabolism in breast muscle of Chongren Partridge chickens. The relative mRNA expression levels of fatty acid elongase 2 (*ELOVL2*), fatty acid elongase 5 (*ELOVL5*), fatty acid desaturase 1 (*FADS1*), fatty acid desaturase 2 (*FADS2*), fatty acid synthase (*FAS*), 3-hydroxy-3-methylglutaryl-coenzyme A reductase (*HMGCR*), lipoprotein lipase (*LPL*), sterol regulatory element binding protein-1 (*SREBP-1*), and carnitine palmitoyltransferase-1α (*CPT1α*) were normalized using *β*-actin as an internal control. ^a,b^ Means in the same row with different superscripts differ significantly (*p* < 0.05). CON, Control; LPCPs, low-dose polysaccharides extracted from *Pogostemon cablin*; MPCPs, medium-dose polysaccharides extracted from *Pogostemon cablin*; and HPCPs, high-dose polysaccharides extracted from *Pogostemon cablin*.

Transcript expressions of genes related to lipid metabolism in breast muscles are shown in Figure 4. Diets supplemented with PCP increased the transcriptive abundance of *FADS2* (*p* < 0.05), but did not affect the transcript abundances of *ELOVL2* (fatty acid elongase 2), *ELOVL5*, Fatty acid desaturase 1 (*FADS1*), 3-hydroxy-3-methylglutaryl-coenzyme A reductase (*HMGCR*), and carnitine palmitoyltransferase-1α (*CPT1α*, *p* < 0.05). The transcript abundances of *FAS* and sterol regulatory element binding protein-1 (*SREBP-1*) were increased by LPCP and MPCP but were not affected by HPCP compared to CON (*p* < 0.05). The transcript abundance of lipoprotein lipase (*LPL*) in MPCP was higher than that in CON and HPCP, which was not a significant difference compared to LPCP (*p* < 0.05).

## 4 Discussion

The functional activity of polysaccharide is usually closely related to their chemical properties, such as molecular weight, monosaccharide composition type and proportion, and the characteristics of glucoside bond (14). Understanding the chemical and physical properties and biological activity of polysaccharides is the basis of polysaccharide application, which will be helpful for its multifunctional application. In this study, the total carbohydrate content of polysaccharide was 76.17 ± 0.23%, the content of uronic acid was 19.92 ± 0.42%, and the content of protein was 1.24 ± 0.07%. The molecular weight is 63.17 kDa and 8.99 kDa, and it was composed of arabinose, galactose, glucose, and glucuronic acid in a molar ratio of 0.196:0.249:0.451:0.104. In general, the antioxidant activity of polysaccharides is proportional to its uronic acid content, and polysaccharides with more arabinose and galactose content have better antioxidant effects (15, 16). Therefore, the excellent biological activity of PCP may be due to its high content of uronic acid, galactose, and arabinose.

*Pogostemon cablin* displays anti-inflammatory, antioxidant, gastrointestinal protective activities, promotion of immune response, and other functions (17–20). *Pogostemon cablin* is a natural plant extract of traditional Chinese medicine. In the current study, dietary supplementation with PCP increased FBW, ADG, and improved feed efficiency, and MPCP had the best effect among the three PCP groups. Some studies found that dietary polysaccharides had the potential to enhance growth performance of poultry. Li et al. (21) reported that the dietary polysaccharides from *Yingshan Yunwu* tea had significant effects on ADFI and ADG of broiler chickens. Ao and Kim (22) demonstrated that 0.02 to 0.04% *Achyranthes bidentata* polysaccharides improved BW gain and feed efficiency. Thus, these findings indicated the applicative feasibility of PCP on growth promoters and high-quality meat production in chicken.

Meat color, pH, and shear force are the major factors for evaluating meat quality. Meat color is an important index that directly reflects meat quality. Studies have shown that consumers prefer pinkish chicken over yellow ones (23). This study showed that a diet supplemented with PCP significantly improves meat color with lower L^∗^ values, b^∗^ values, and higher a^∗^ values of breast muscle. Zhao et al. (24) agreed with our results that dietary phytosterol supplementation decreased L^*^ values in muscle. Liu et al. (25) also reported that dietary natural capsicum extract decreased muscular L^*^ value. These results suggest that dietary PCP is an effective feed additive to improve the meat quality of chicken.

IMP and GMP are important indexes for evaluating the umami taste of meat (26). In the current study, dietary PCP significantly increased the content of GMP, which means that PCP may make chicken tastier. Li et al. (27) found that a diet supplemented with polysaccharides extracted from *Yingshan Yunwu* tea increased IMP and GMP. Moreover, the enhancement of chicken meat flavor might further lead to improved amino acid metabolism and free amino acid levels (28, 29), which is in accordance with the results of this study.

The amino acid profile is a crucial factor in meat quality evaluation, and free amino acids are regarded as important indicators influencing flavor traits such as the taste and fragrance of meat (21). In the present study, we found that the amino acid profile of breast muscles was significantly altered by the diet supplemented with 400 mg/kg PCP. Specifically, our results indicated that chickens fed with the 400 mg/kg PCP diet increased the concentration of majority free amino acids such as alanine, aspartate acid, as well as total FAAs, EAA, and AA in breast muscles. These results suggest that dietary PCP, especially 400 mg/kg of PCP, improves meat flavor and nutritional value by altering the amino acid profile and increasing the content of total FAAs, EAA, and AA.

Studies have shown that MDA is produced as a result of lipid peroxidation, and its content directly reflects the degree of oxidative damage (30, 31). In the current study, diet supplemented with PCP decreased the content of MDA in meat, which was consistent with previous work that dietary inclusion of CE and chlortetracycline could enhance antioxidant enzyme activity of SOD and decrease MDA concentration (32). Zhang et al. (33) also demonstrated that MDA levels in broilers were significantly decreased by dietary resveratrol supplementation.

The fatty acid composition determines the nutritional value and oxidative stability of muscle and also plays an important role in meat quality and the acceptability of meat products (34). PUFA is an important component of foods, and foods with higher levels of PUFA may reduce the risk of metabolic syndrome and inflammation (35, 36). In the current study, the fatty acid composition of chickens fed with the PCP diet was changed and the content of c14:1, c16:1, c22:2, and total MUFA was increased. Alonso et al. (37) found that high PUFA levels increased lipid susceptibility to muscle peroxidation. The results of this study showed that the content of PUFA in meat of MPCP was the highest, which may be due to the excellent antioxidant activity in the body of PCP in the medium dose, which inhibited the oxidation of PUFA. To further investigate the effects of PCP on lipid metabolism in muscles, we examined the transcript expression of genes related to lipid metabolism. Results of this study indicated that dietary PCP generally increased expressive abundances of *FADS2*, *LPL*, and *SREBP-1* of breast muscles in broilers. Jing et al. (38) found that *FADS1*, *FADS2*, *ELOVL2,* and ELOVL5 genes were upregulated with a decrease of 18:2 n-6 (LA)/ALA ratio in diets. Gou et al. (39) also found that *FADS1*, *FADS2*, *ELOVL2,* and *ELOVL5* genes were upregulated by dietary LO with SI supplementation in the birds. These results indicate that dietary supplementation of PCP changes the composition of fatty acids in breast muscle by regulating the expression of related genes.

## 5 Conclusion

In conclusion, the present study demonstrated that a diet supplemented with PCP increased the FBW and ADG and decreased F/G; especially, 400 mg/kg PCP increased flavor substance and antioxidant capability and improved amino acid profile and fatty acid composition. Further, these changes may be mediated through the regulation of the expression of genes related to lipid metabolism.

## Data availability statement

The data analyzed in this study is subject to the following licenses/restrictions: Limited data access or request required (still available). Requests to access these datasets should be directed to YT, tangyantian1028@163.com.

## Ethics statement

The animal study was approved by Animal Care and Use Committee of Jiangxi Agricultural University (JXAUA01). The study was conducted in accordance with the local legislation and institutional requirements.

## Author contributions

YT: Conceptualization, Writing – original draft. SC: Methodology, Writing – review & editing. LC: Formal analysis, Writing – review & editing. KO: Formal analysis, Writing – review & editing. HC: Formal analysis, Writing – review & editing. WW: Conceptualization, Funding acquisition, Writing – review & editing.
